# Supplementary material for: Interaction between PHB2 and Enterovirus A71 VP1 Induces Autophagy and Affects EV-A71 Infection
Source: Viruses. 2020 Apr 8;12(4):414. doi: 10.3390/v12040414 (PMC7232526; doi:10.3390/v12040414)
Supplement: Supplementary file 1 [file viruses-12-00414-s001.pdf]

viruses

MDPI

Table S1. Primers for amplifications of the full-length and fragmented VP1 genes. To clone the genes into the plasmid pEGFP-N1, the 5' end of the forward primers and the reverse primers were added the sequences of protected bases and restriction sites (forward primer: CCGGAATTC; reverse primer: CGCGGATCC), and the Kozak sequence (GCCACCATG) and the flag-tag sequence were added to 5' end of the forward primers.

Name	Sequence (5'to3')	Position of the amplified fragments in VP1
VP1-F	GGAGATAGGGTGGCAGATGT	1–891
VP1-R	GAGAGTGGTGATTGCTGTACG	
VP1 (1)-F	GGAGATAGGGTGGCAGATGT	1–150
VP1 (1)-R	AGCGGCTTGGAGTGCTGGGACTTTA	
VP1 (2)-R	GAGATTGGAGCATCATCAAA	151–300
VP1 (2)-R	TGTGCCTTCAAGAGGGAGGTCTATC	
VP1 (3)-F	ACTAACCCGAATGGTTATGC	301–450
VP1 (3)-R	CAACTGTGGGACAACTTCCCCGGTG	
VP1 (4)-F	CTCCAATATATGTTTGTGCC	451–600
VP1 (4)-R	AGCACTCGCGGGTGACATAAATGGA	
VP1 (5)-F	TATCAATGGTTTTATGACGG	601–750
VP1 (5)-R	CCTAATCACTAAAGGGTACTTGGAC	
VP1 (6)-F	ATTTACATGAGGATGAAGCA	751–891
VP1 (6)-R	GAGAGTGGTGATTGCTGTACG	
VP1 (1-5)-F	GGAGATAGGGTGGCAGATGT	1–750
VP1 (1-5)-R	CCTAATCACTAAAGGGTACTTGGAC	

viruses

MDPI

Table S2. Primers used for RT-qPCR analysis.

Name	Sequence (5'to3')
EV71-F	CCAGAAGAATTTTACCATGAAGTTGT
EV71-R	AGGGCTCTGCTCATACTATC
EV71-Probe	CAGACGGGCACTATACAGGGAG
GAPDH-F	GATTCCACCCATGGCAAATTCCA
GAPDH-R	TGGTGATGGGATTTCCATTGATGA
PHB2-F	CGTGGAAGGCGGTCATAGAG
PHB2-R	GGGGATCCTGAAGTGAAGGC
ATP6AP2-F	CCATGGCTGTGTTTGTCGTG
ATP6AP2-R	TCCGCTCTCCTGGTATAGGC

viruses

MDPI

Table S3. Mass spectrometry analysis of proteins contained in band 1 (PSMs > 10).

Protein Group ID	Proteins	Unique Peptides	Peptides	PSMs	Group Description
42	3	23	23	27	Desmoplakin
22	5	8	17	27	Keratin, type I cytoskeletal 14
59	5	8	9	26	Heterogeneous nuclear ribonucleoproteins A2/B1
23	1	15	15	19	Hornerin
90	5	7	13	19	Keratin, type I cytoskeletal 16
7	2	1	5	15	Keratin, type II cytoskeletal 1b
13	3	11	11	14	Serum albumin
27	6	1	2	13	Isoform 3 of keratin, type II cytoskeletal 80
57	2	11	11	12	Junction plakoglobin
78	4	4	9	11	Keratin, type I cytoskeletal 17
96	7	1	2	11	Heterogeneous nuclear ribonucleoprotein A1
41	2	8	8	10	Desmoglein-1
69	2	7	7	10	Suprabasin
49	9	2	6	10	Actin, cytoplasmic 1

viruses MDPI

Table S4. Mass spectrometry analysis of proteins contained in band 2 (PSMs > 10).

Protein Group ID	Proteins	Unique Peptides	Peptides	PSMs	Group Description
19	2	2	11	30	ADP/ATP translocase 3
79	2	3	11	29	ADP/ATP translocase 2
42	1	17	17	23	Hornerin
34	5	2	3	23	Heterogeneous nuclear ribonucleoprotein A0
64	9	1	17	23	Keratin, type II cytoskeletal 6B
114	2	2	9	21	ADP/ATP translocase 1
90	3	17	17	20	Desmoplakin
112	2	12	12	20	40S ribosomal protein S3
159	1	8	8	20	40S ribosomal protein S3a
144	3	7	7	18	40S ribosomal protein S4, X isoform
199	5	5	10	16	Keratin, type I cytoskeletal 16
208	8	2	3	16	Heterogeneous nuclear ribonucleoprotein A1
146	5	6	6	15	Ribose-phosphate pyrophosphokinase 1
122	1	8	8	14	60S ribosomal protein L7a
211	2	7	7	13	Phosphate carrier protein, mitochondrial
77	2	10	10	12	Prohibitin-2
186	2	9	9	12	Mitochondrial 2-oxoglutarate/malate carrier protein
38	5	3	9	12	Keratin, type I cytoskeletal 14
6	3	6	6	11	60S acidic ribosomal protein P0
176	5	5	8	10	Keratin, type I cytoskeletal 17

Table S5. Mass spectrometry analysis of proteins contained in band 3 (PSMs > 10).

Protein Group ID	Proteins	Unique Peptides	Peptides	PSMs	Group Description
19	3	3	11	37	ADP/ATP translocase 3
72	2	4	12	35	ADP/ATP translocase 2
103	2	12	12	18	40S ribosomal protein S3
71	2	13	13	16	Prohibitin-2
149	1	8	8	16	40S ribosomal protein S3a
139	3	3	7	16	Ribose-phosphate pyrophosphokinase 1
38	1	9	9	15	Hornerin
137	3	9	9	14	40S ribosomal protein S4, X isoform
12	5	1	6	14	Keratin, type II cytoskeletal 1b
18	1	10	10	12	UPF0568 protein C14orf166
147	1	8	8	12	40S ribosomal protein S2
113	1	8	8	12	60S ribosomal protein L7a
187	2	2	6	12	Isoform 2 of Ribose-phosphate pyrophosphokinase 2
204	4	1	1	12	Ig kappa chain V-II region RPMI 6410
82	3	10	10	11	Desmoplakin
189	2	9	9	11	Isoform 2 of ELAV-like protein 1
22	3	7	7	11	Serum albumin
95	13	7	7	11	Actin, cytoplasmic 1
202	9	5	5	10	Heterogeneous nuclear ribonucleoprotein A1

viruses

MDPI

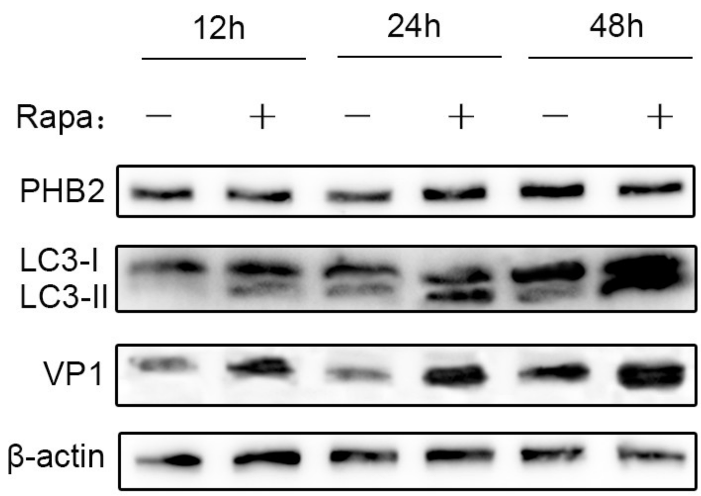

Figure S1. Rapamycin treatment increased autophagy and VP1 expression. RD cells were pretreated with rapamycin and infected with EV-A71 (MOI = 1). At different time points after infection, proteins were detected by Western blot analysis.
